# The Endoscope, and Its Application to the Diagnosis and Treatment of Urinary Affections

**Published:** 1867-11

**Authors:** A. J. Desormeaux


(*Continued from page 464.*)

the autopsy. In the other parts, the pus which could not be washed away by the injections hides from you the state of the membrane; but the presence of this purulent layer, which we also cannot detach from the end of the sound, is already a proof of the ulceration of the bladder, and a lesion very grave upon its surface.

Before quitting this subject I will call your attention to a patient which we have lately examined. From his entrance he presented already some time since, some symptoms of cystitis; the micturition was accompanied and, above all, followed by pains; the urine contained pus; and he had had a little hematuria. We suspected a calculus, but the sound did not show any foreign body. The endoscope showed us the vesical membrane of a deep red, surrounded by an abundant network of vessels of a more lively red, the border of the prostate was red; but that which was most notable, was a development of round red points, larger than granulations and more regular than flesh buds. I had some difficulty to satisfy myself in regard to these growths, however, their appearance leads me to suppose that they are simply inflammatory.

The following confirms this diagnosis, for after some time of repose, from baths and antiphlogistic treatment, all the symptoms disappeared, and then, on a last endoscopic examination, the bladder and the prostate had recovered their normal appearance; the prostate, in particular, had again become smooth and even, as in its natural state.

We have already had occasion to speak of the engorgement of the prostate, and also of urethral ulcerations, which are sometimes the cause, but we have said nothing of the endoscopic character of the swelling which follows.

The engorged prostate can acquire a considerable volume; and, as you know, makes a lump in the cavity of the bladder. Its volume can be appreciated by the depth to which it is necessary to pass the sound, in order to penetrate into the organ and touch it through the rectum; its prominence in the bladder and its measure, with sufficient precision, may be known by means of the catheter of M. Mercier, but through the endoscope we can sometimes know more, the disposition of it. A first particularity which strikes the observer is, that, in withdrawing the sound, instead of seeing the border of the prostate rise gradually into the field of the instrument, it follows the surface of the glass in the form of a curtain; we see it from the bottom to the top, formed at the end of the instrument, like the action of the sucker of a pump, it relieves itself after being pressed. If the instrument rests upon the middle portion of the lobe, which was thus placed before the sound, it turns from behind and appears difficult to free the passage. It seems like a cover or lid which appears to shut upon itself and raises itself. When we have seen this appearance it is impossible to confound it with the normal state.

Whilst afterwards following the contour of the vesical orifice, we find that its inferior part, in place of a crescent with a superior cavity, presents a convex line towards the top, and, in thus following the border of the middle lobe, where the prominence is situated which it forms in the bladder, the middle lobe, engorged, offers, in general, a contour uniformly round, whilst it is not an organic affection. However, upon a patient who had been operated on several times, by a section of the prostatic valvule, and who had had an abscess of the prostate opening into the bladder, I have found an irregular prominence with deep holes, which resembled a hard damaged substance, which the organ had formed in the hypertrophied lobe by the cicatrization of the abscess.

Since the time that I have applied myself to the endoscope, I have not had the occasion to apply it to tumors of the bladder, properly so-called; but, finally, we had in our wards two cases of cancerous degeneracy of this organ and of the prostate. These two patients had experienced dysuria, and had entered into the hospital because they had been seized with complete retention; their urine was very turbid, and they had considerable hematuria, particularly one of them. Rest and treatment had mitigated the pains of the retention, and then certain specific pains manifested themselves, which, above all, had sometimes a freely lancinating character. Upon one of these patients, the sound had not detected any tumor, only it sunk very deeply before it arrived in the vesical cavity. On examining with the endoscope, it was impossible to perceive the border of the prostate and the orifice of the urethra, it was found that only one surface continued itself from the prostate to the "bas-fond" of the bladder, and passing up very high into the organ; this surface, of a yellowish white, with the vessels sufficiently developed, formed nipples, large and very prominent, and presented at a place ecchymoses of a deep red, which had all the appearance of an encephaloid mass. It appeared to form a large prominence badly confined or limited, in a manner, although the sound not being well free in the bladder, could not exactly circumscribe a tumor. Upon the other patient, catheterism did not discover anything in the bladder, except some inequalities, which were situated around the fleshy columns; but at the neck there was found, by the aid of the endoscope, a prominence, considerably large, of the middle lobe of the prostate; you have seen all the observable face of the bladder strewn with soft vegetations, of a reddish yellow, forming small teats or nipples, and presenting the same aspect and coloration of encephaloid vegetations. The prostate makes an inconsiderable prominence in the bladder, it is true, but presenting the same aspects as in the vesical wall.

The sediment in the urine of these patients, and examined by the microscope, contains, besides globules of pus, great nut-like cellules, which presented the character of cancerous cellules. These cellules were very numerous in the urine of the second patient.

The group of symptoms and, above all, their persistence, the cachectic appearance which these patients had acquired by degrees, then likewise as the local symptoms appeared a little calm, and the microscopic examination of the urine could give some strong presumptions; but without the endoscope, there is but little degree of certainty in the diagnosis, and I wish you to notice that this diagnosis had been made in the two cases, after the results of the endoscopic examination, before the symptoms had furnished suspicion of the nature of the evil, and whilst the tint of the skin did not yet betray the development of the cachexia.

If we consider how few means we have in order to assure ourselves of the nature of a tumor, which the sound has reconnoitred in the bladder, how little certitude of differential signs of these tumors between themselves, so much so that the cachexia is not evident, and with danger of attack from a cancer which we cannot completely destroy; we are really astonished at the hardihood of deciding either for the ecraseur or for the "arracheur."

You will be convinced, I think, that the endoscope has already rendered a grand service to the surgeon and the patient, if it prevents the performing an operation on a cancer, of which the result must be a speedy death.

It rests with us to speak to you, gentlemen, of an alteration which is the consequence, for the most part, of affections of this viscera. You know that whilst the bladder empties itself with difficulty, in consequence of some obstacle in the course of the urine, it happens, about a certain time, that the bundle of muscular tunic becomes hypertrophied and makes a prominence upon the internal surface of the organ, the mucous membrane being enfolded in their intervals, and from it results that we call "bladders with columns," and "bladders with cellules."

In this case, these columns hinder the ordinary catheter, whilst its point passes on the mucous membrane, giving the sensation of prominences which it meets, but it is necessary for this that the columns be already sufficiently voluminous; but, still further, other prominences can be diagnosed in the bladder, from tumors, from cancerous vegetations, or otherwise, also this—mistakes are not very rare, and we arrive at the belief, from columns in the smooth bladder, of mistaking columns for tumors, and reciprocally.

The endoscope enables us to avoid these errors, and, therefore, they have become more rare; it shows the columns under the form of "cordons arrondes," as you see them in the autopsies, with their more salient points very clear, and the adjoining parts in the shade. Whilst these prominences are large, their two borders do not show themselves simultaneously in the field of the instrument, and it becomes necessary from small movements to measure the larger. It is easy to follow in the way of their length, and then we see them united, then divide themselves, intercepted between them some depressions in the mucous membrane or the veritable cellules.

Whilst we can thus reconnoitre their form, it will be impossible to confound them with any other thing, so much the more as they present the general color of the mucous, whether it be sound or inflamed, and which is not the same, frequently, of tumors, which have in the bladder, as elsewhere, their particular coloration.

We will terminate this exposition of the results of the endoscopic examination of the urinary passages, by leading you to a knowledge of the means which can be taken for the exploration of the vesical calculi. In order that this exploration may succeed, it becomes necessary to direct the axis of the instrument in the direction of the foreign body. As soon as this condition is obtained, it is easy to verify the color of the calculus, and, from certain movements, to reconnoitre the aspect of its surface, its smoothness, its nipple-like prominences, irregularities, or unevenness, if we carry the end of the sound upon the contour of the calculus, or can get an exact idea of its form, and likewise appreciate sufficiently well its volume; however, for persons who have not yet the proper arrangement of the instrument, the dimensions of the object appear, frequently, a little larger than they really are. But this error is very far from attaining the extent of those to whom it is exposed by the other means of exploration, and, in a practical point of view, it is of little importance. For, if the exact measure is necessary, it is easily obtained. For that, it suffices to mark well how many times the field is limited by the extremity of the sound, how many times it finds itself contained in each of the principal diameters of the calculus.

The interior diameter of the sound being known, we can conclude on the dimensions of the calculus with a quite sufficient exactitude. It is very rare that the surface of the stone be sufficiently smooth, so as not to furnish points of observation. It is thus that our designer, M. Lackenbauer, has been able to measure the different calculi which he has represented, and of which you have seen the designs. These measures have been proven just, upon certain ones after their extraction, and upon others whilst they have been taken in the "litholabe," in order to be crushed.

Before going much further, I will give you some details in regard to the calculi, from which I have made designs after nature, in the bladder of the patients, before they were operated on. One represents a group of four calculi, which brought a sick man into my service in the month of February, 1862.

In the month of October preceding, he had consulted a surgeon, who had proved the presence of some gravel, and had scraped some debris into the grasp of the litholabe. Some days after, the surgeon introduced the instrument anew, and not finding any fragments, declared the patient cured.

From the first examination by the aid of a sound, I proved the presence of a small calculus, very moveable; such was also the diagnostic of a confrere, well versed in catheterism. Some days after, the endoscope showed us the four calculi, such as they have been designed by M. Lackenbauer, on the 9th of March. The 16th of March, a new examination enabled this artist to design them in the two groups, in each of which one of the small calculi is found hidden. The endoscope had permitted, at first, to verify, at the "bas-fond" of the bladder, a large depression, very deep, but in which the calculi had collected, so much that the rest of the vesical surface was covered over by the fleshy columns very much developed. After two seances of lithotrity, the endoscope proved the presence of fragments. It became necessary to seek these fragments, which the bladder could not get rid of, on account of the commencement of paralysis and hypertrophy of the prostate.

Some days later, the patient, who did not suffer any more, was taken with stoppage or extreme difficulty of breathing, and succumbed to an old disease of the valves of the heart.

The autopsy showed the depression of the "bas-fond" of the bladder and the columns which the endoscope had discovered. At the first seance of lithotrity, in seeking to measure the calculus, I have always found by a very great scattering of the branches of the litholabe the greatest of the diameters transversely of a large calculus. This is the diameter which finds itself in a sence-antero-posterior.

After the configuration of the calculus, it appears evident that they are not fragments of a calculus greater than had been broken by the first surgeon.

It is very evident that the view of a calculus will add something to the certainty that the touch will give to its presence. But you may say that there are some causes which render very difficult the finding a stone in the bladder. Thus, whilst it is small and very mobile in a very large bladder, in a way it flies before the sound, without giving place to a sensible shock. In this case, the sight cannot leave any doubt, and it suffices by a *coup d'œil* thrown upon the Fig. 5, to convince that a gravel cannot escape, provided that the sound can be directed towards it. The rugose state, smooth, broken of the surface of the calculus, display themselves more surely than by means of the sound, and there is no other means of appreciating the color, which often permits you to presume upon the composition.

Simple catheterism gives us but a few precise ideas of the dimensions of the calculus; the litholabe likewise; but it cannot comprehend it in one sense, neither can it give the measure of all the diameters, for it is known that each calculus affects, in general, always the same position, determined by the relation which exists between its form and that of the bladder. The endoscope, on the contrary, shows all the diameters, so much more than one can accomplish with the aid of the sound, by displacing the calculus and making it present in different positions.

The number of the calculi is, again, an important condition for the treatment, and difficult to know by the ordinary means. Beyond doubt, in seizing a calculus in the grasp of a "brise pierre," and in seeking to explore the "bas fond," we can assure ourselves that there exists a second. But, if there are two, three, four, or more, we can perceive them, so long as the endoscope permits us to count them.

I have not had other occasions of observing multiple calculi, but upon the cadaver it is easy to perceive a great number of stones introduced expressly into the bladder. If it is impoıtant to verify the presence of a calculus, and the different particularities which it is necessary for us to know, it is just as important for us to know that it does not exist as it is that it does. This error, however, can present itself, it has had a place and it still remains in the annals of medical science, some cases of patients having been cut without having a stone. Authors indicate many causes of this terrible error, the principal ones are, hard matter contained in the rectum and pressing against the base of the bladder, it appears to me difficult that in this case the contact should be sufficiently rough to deceive a well practised hand; it is not the same of an osseous tumor of the pelvis making a prominence in the bladder, and which can give, well covered by their tunics of this organ, a sensation of hardness capable of deceiving the surgeon, or very much to embarass him.

A fleshy column, resisting, has also been noted as a cause of error; it has been well objected, too, that it had not a hardness comparable to that of the stone, but I can tell you by experience that it is from cases of induration of the vesical walls when the fleshy columns are sufficiently hard, that it is very difficult to distinguish the sensation which we experience in touching with the end of the sound from that which is caused by contact with a calculus.

In addition to this, sometimes the mucous membrane, in old affections of the bladder, becomes encrusted with lithic matter. Then you have the resistance of a hard body and the rude touch of a stone. In this case, it is almost impossible, by the aid of the sound, to assure ourselves of the truth.

It can be objected, perhaps, that the question can be solved by means of the litholabe, but, very often, it leads to the belief of an adherent calculus, and the manœuvres by which it seizes the vesical prominence and presses it in the grasp of the instrument, in seeking to displace it, cannot certainly be without danger. I have often had patients who presented this disposition, and whom I have observed at the Hotel Dieu, whilst I was surgeon of the Central Bureau. The sensation was that of a calculus. I requested Ph. Boyer and Leroy (d'Etiolles) father, who had come into the service, to give me their advice, but after a very attentive examination it was impossible for us to decide on a certain diagnosis, and we could not decide on anything, if it was not that the sick man being in a very bad condition to be operated on, we should have decided on it. Some days later, the autopsy showed us the alterations which I have spoken to you of. If I had had the endoscope, you do not doubt, I think, after all that you have seen in this amphitheatre, that the question could have been decided while the patient was living.

One question which presents itself very often, and which is of no little importance, is that of the adherence or enchainment of the calculi, upon which often depends the mode of operating. One suspects this enchainment whilst the stone is always found at the same point which we can displace, whilst the instrument glides smoothly over the surface without moving it; but it is rare that we arrive at a certainty without opening the bladder. If we survey the observations of united or enchained stones you will perceive that often this disposition had not been perceived, except during the operation, whilst the forceps could not seize the calculi, or to hold them after having seized them.

From it results a very embarrassing position, when the surgeons have often given proof of *sang froid*, of ability, of promptitude, and of decision. But if sublime has been the success, it will be of more value in operations less difficult, and operators will prefer to know in advance the difficulties they have to encounter.

By means of the endoscope, we can assure ourselves of the disposition of the calculus, and of the causes which render it immoveable. In effect, in this case, one finds around the calculus some clots of mucus, of prominences, of vegetations, which fix it in its place. I have not yet made sufficient observations in order to give you a description of the different modes of enchainment, but the following observations can supply them in part:—

The first, is that of a calculus, which I have examined and made a design of, from the Hospital Saint Antoine, in the service of Prof. Jarjavay. The patient presented the rational signs of a calculus, which M. Jarjavay found, in fact, by catheterism. But this calculus was difficult to reach, the litholabe could not seize it; at the same time, it could not be determined whether it was fixed or moveable. However, M. Jarjavay suspected that it was fastened, but not being fully satisfied, he requested of me the aid of the endoscope. The endoscope resolved the question completely. The calculus was, in effect, retained between the projections of the bladder; the right extremity appeared to be free, the left extremity and the superior border being surrounded by a ridge of the mucous membrane, and the inferior border, finally, is in part hidden by a veritable tumor, upon which is noticed an ecchymosis, which appears to result from the pressure of the grasp of the litholabe, in the forceps made to seize the calculus, proving that if the expert surgeon had insisted upon his tentatives, it would have resulted in a wounding of the vesical mucous membrane. The endoscope would have prevented, beyond all question, a similar accident.

The second observation was furnished me by M. Houel, temporarily in charge of the surgery at the hospital clinics, during the last vacancies. The irritation of the bladder in his patient prevented me from making a design of this calculus, but M. Houel has earnestly requested me to give the following observation, in which the autopsy confirmed the results of the endoscopic examination:—

*Calculus fixed to the Bladder—Examined with the Endoscope.*

J., aged 58 years, a paint merchant. This man was very large and strong, but he appeared to be suffering much. He said to us, that for many years he had suffered much from the bladder. He is obliged to urinate very often; the micturition is painful and very difficult.

Whilst he walked, he felt a feeling of weight in the perineum; he often had pains of the kidneys. For five or six months, he had lost his appetite and had become thin and poor; his urine was very turbid and deposited a sediment of mucus, very thick, sometimes mixed with blood. Never, in addition to what we have said, had this patient seen his jet of urine suddenly arrested, except to return some moments after.

Whilst a sound is introduced into the bladder, mark that which is observed: the canal of the urethra is free and allows the sound to pass easily. One time that it penetrated into the bladder, we could reconnoitre a bladder of reasonable dimensions, with columns, a bladder very smooth. If we carry the pavilion of the sound a little higher to the left of the patient, we perceive that the end of the sound is arrested by a stone, which, by consequence, ought to be found at the base of the bladder and to the right. In introducing a lithotrity instrument, it is impossible to seize the stone; we touch it but it will not fall into the grasp of the instrument, and we cannot seize it.

This examination, repeated several times, induced M. Houel to diagnose a fixed calculus of the bladder, although M. A. Richard, in sounding this patient, believed the calculus free, because the end of the instrument dragged the calculus and the bladder. M. Houel solicited M. Desormeaux to dissipate the doubt, by means of his endoscope. I will not enter into the details of his mode of action, which is known by all surgeons; I will speak of nothing except the result of the observations.

In manipulating the instrument into its necessary manœuvres, one can see, in turning the instrument in divers ways, a whitish granular body, strewn with small blackish points which appear to be blood. This body, which is no other than the calculus, presents a light or small prominence; and, if we should move the instrument, we arrive at the edge of the calculus, and we see very distinctly that this white body is surrounded and, in part, covered by a reddish membrane, which is continuous with the vesical mucous membrane.

This examination confirmed, very closely, the diagnosis of calculus fixed in the bladder. In the face of this condition, M. Nélaton had counselled the sub-pubic incision, and M. Houel commenced the operation, when the patient suddenly died.

The autopsy gives the following signs or appearances:

The bladder is not very much changed; the calculus is well situated at the place which the examination had given to us; the calculus is well covered by the bladder upon its borders, but at this level the bladder is a little hypertrophied, likewise, the calculus is strongly held by the bladder; furthermore, the calculus presented an eminence, which penetrated the vesical walls, where it was contained, as in a cellule; the calculus had yet the black points that we have upon the living. One point, sufficiently curious to note, is, that to the naked eye the calculus appeared less voluminous than with the endoscope.

If we resume this lesson, we will find that in the affections of the bladder, the endoscope oftentimes gives more of certainty in its diagnosis than the other means; it permits us to see the state of the organ, to reconnoitre the nature of its tumors, the disposition, the number and the complications of stones, oftentimes better than simple catheterism; better, and, likewise, with less danger, than the "litholabe."

We shall then conclude that if we cannot, in the vesical affections as in the affections of the urethra, proceed directly to operations, it is not less useful to the treatment, in furnishing valuable indications to determine the choice of the method for operating, and to direct the instruments in the operation to be practiced.

